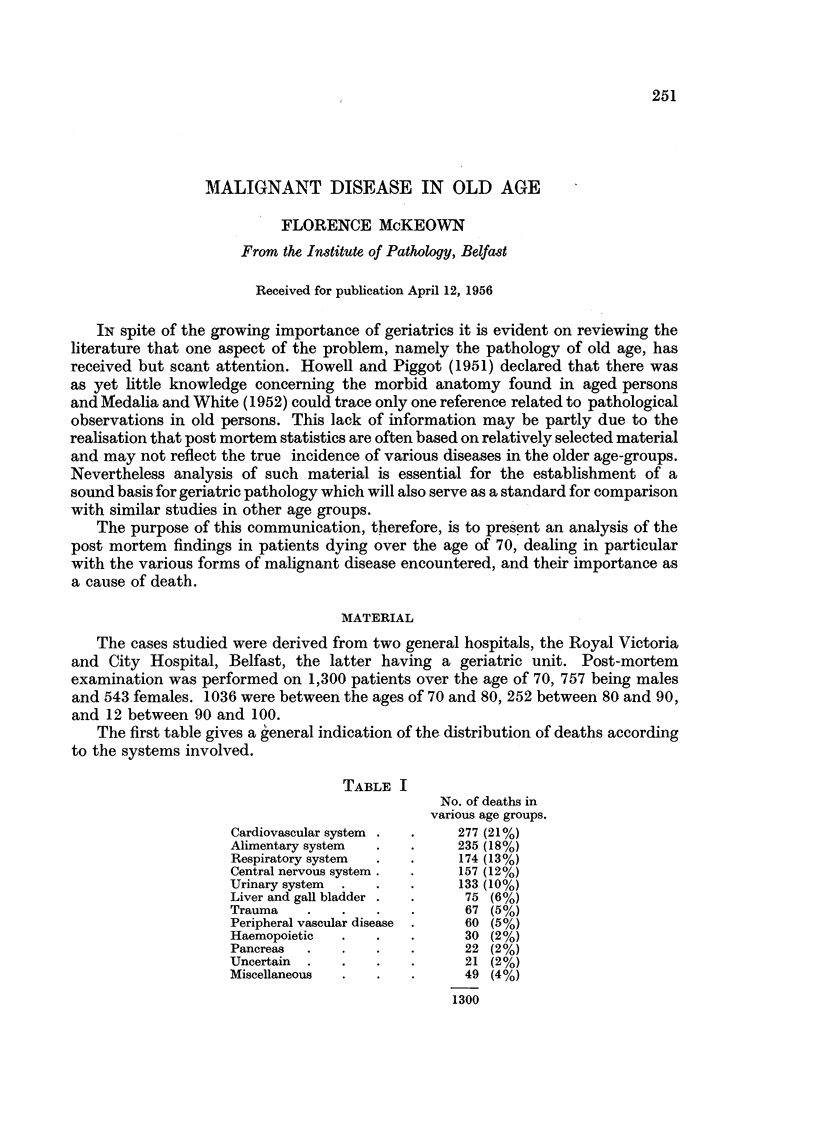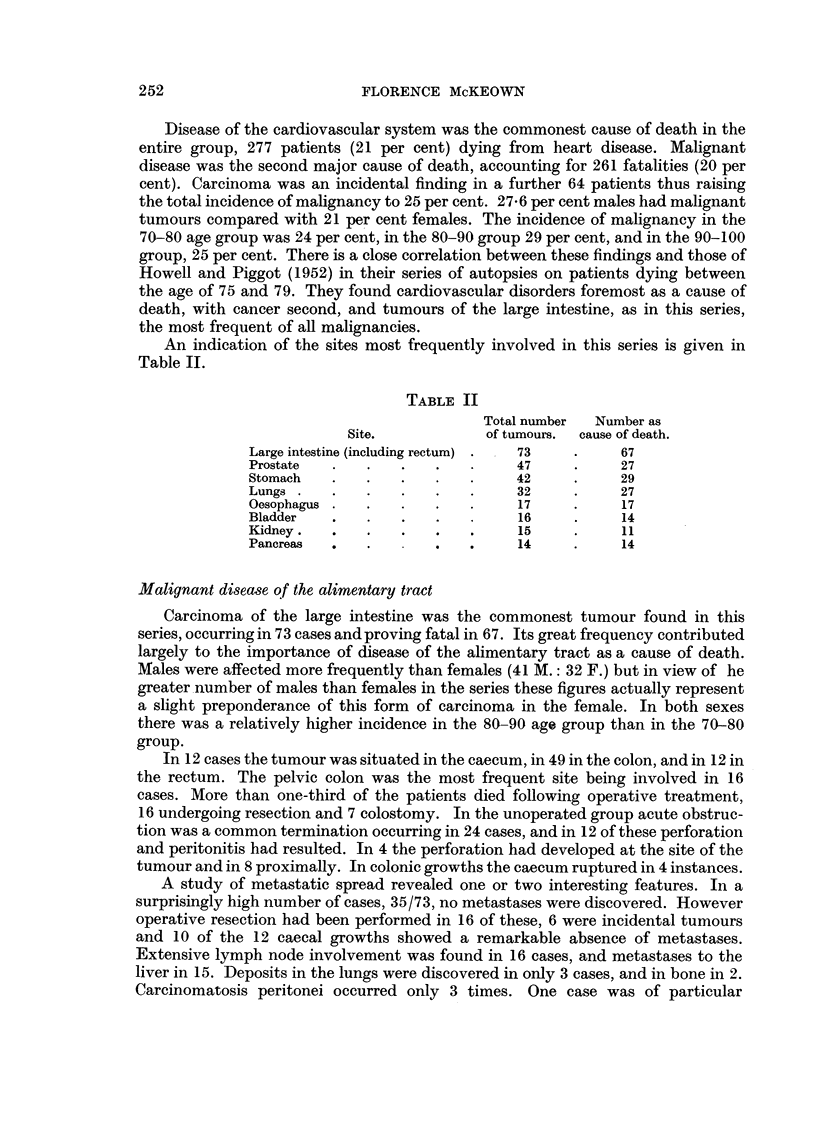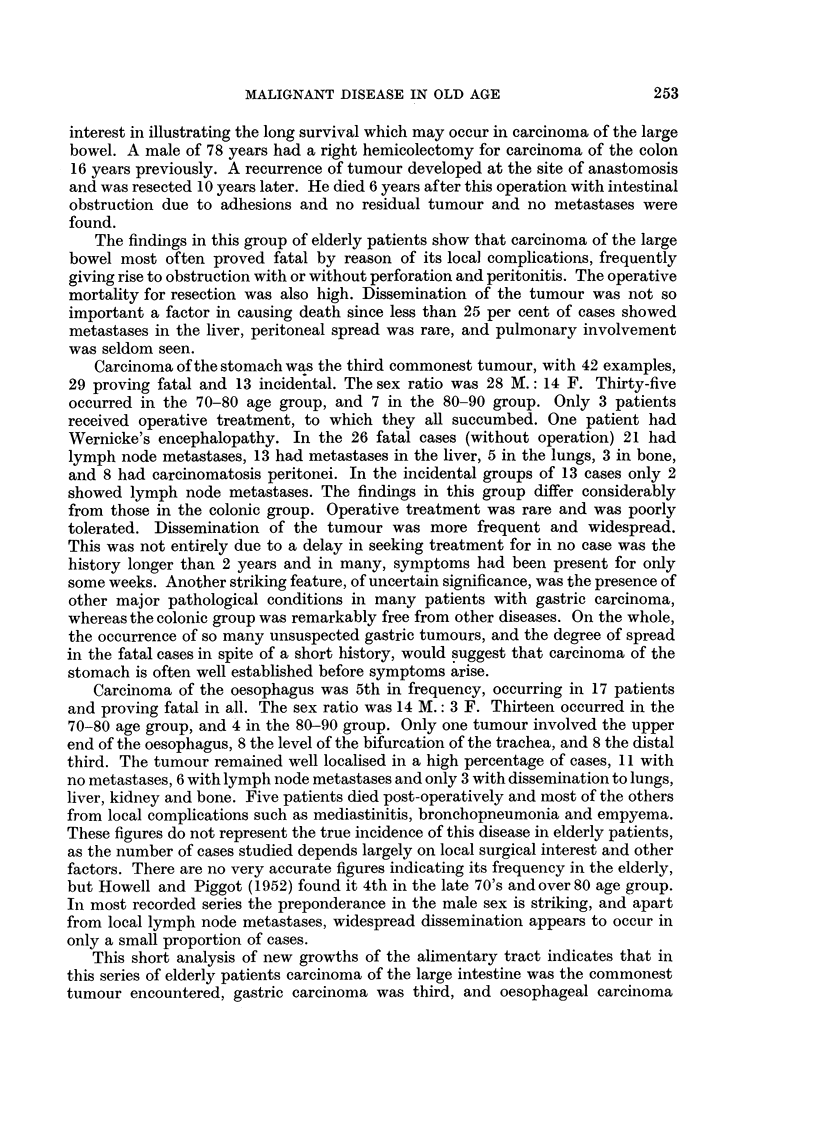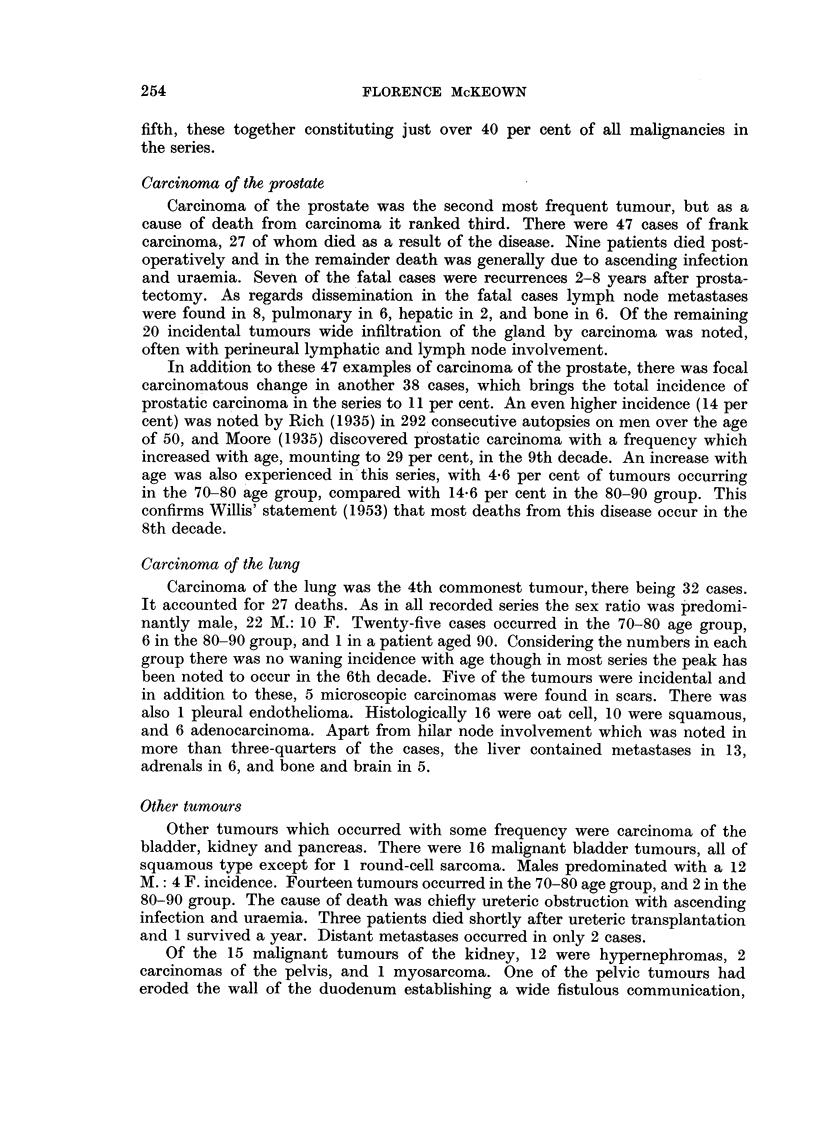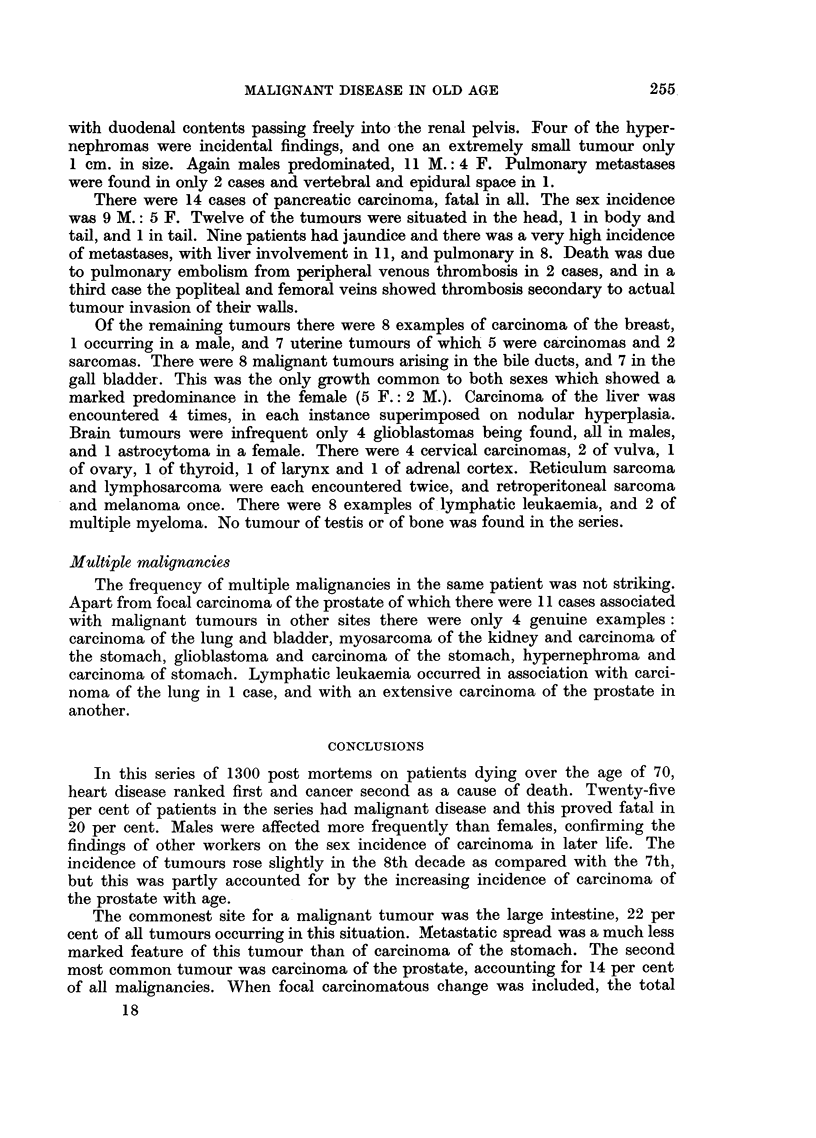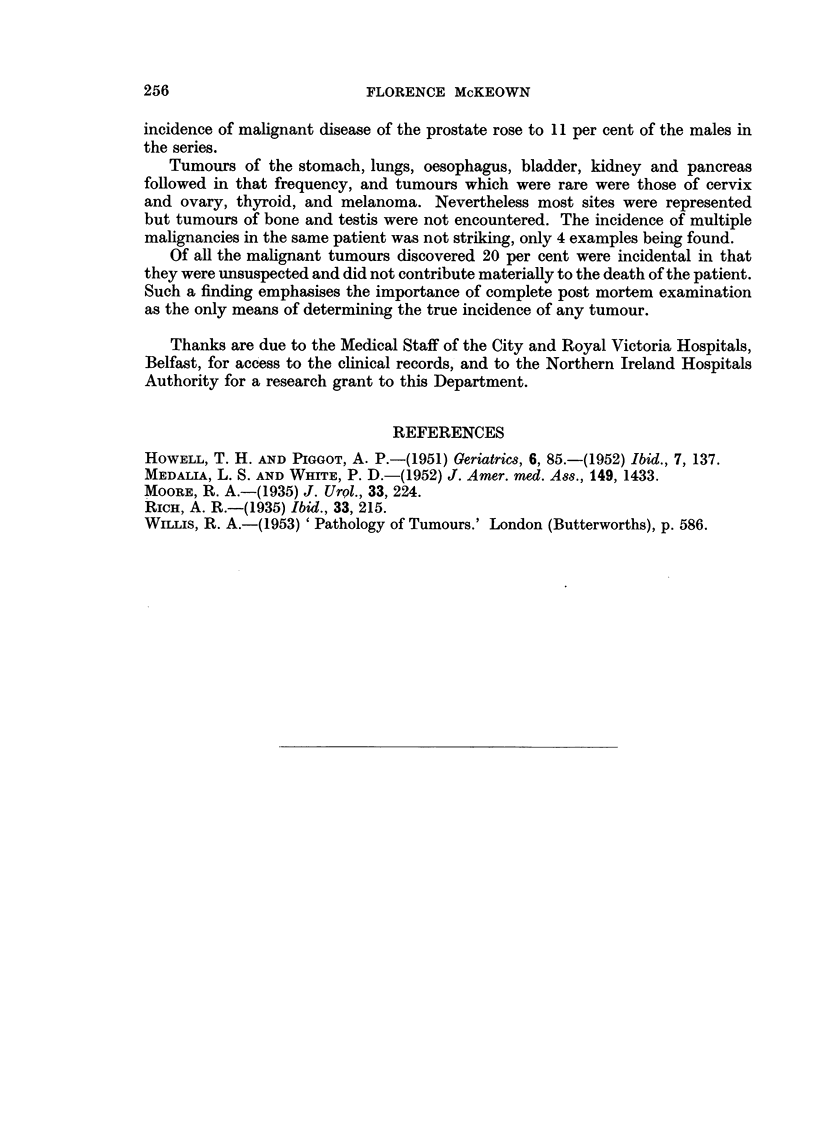# Mtalignant Disease in Old Age

**DOI:** 10.1038/bjc.1956.29

**Published:** 1956-06

**Authors:** Florence McKeown


### MALIGNANT DISEASE IN OLD AGE

FLORENCE McKEOWN

*From the Institute of Pathology, Belfast*

Received for publication April 12, 1956

IN spite of the growing importance of geriatrics it is evident on reviewing the literature that one aspect of the problem, namely the pathology of old age, has received but scant attention. Howell and Piggot (1951) declared that there was as yet little knowledge concerning the morbid anatomy found in aged persons and Medalia and White (1952) could trace only one reference related to pathological observations in old persons. This lack of information may be partly due to the realisation that post mortem statistics are often based on relatively selected material and may not reflect the true incidence of various diseases in the older age-groups. Nevertheless analysis of such material is essential for the establishment of a sound basis for geriatric pathology which will also serve as a standard for comparison with similar studies in other age groups.

The purpose of this communication, therefore, is to present an analysis of the post mortem findings in patients dying over the age of 70, dealing in particular with the various forms of malignant disease encountered, and their importance as a cause of death.

### MATERIAL

The cases studied were derived from two general hospitals, the Royal Victoria and City Hospital, Belfast, the latter having a geriatric unit. Post-mortem examination was performed on 1,300 patients over the age of 70, 757 being males and 543 females. 1036 were between the ages of 70 and 80, 252 between 80 and 90, and 12 between 90 and 100.

The first table gives a general indication of the distribution of deaths according to the systems involved.

TABLE I

| | No. of deaths in various age groups. |
|---|---|
| Cardiovascular system | 277 (21%) |
| Alimentary system | 235 (18%) |
| Respiratory system | 174 (13%) |
| Central nervous system | 157 (12%) |
| Urinary system | 133 (10%) |
| Liver and gall bladder | 75 (6%) |
| Trauma | 67 (5%) |
| Peripheral vascular disease | 60 (5%) |
| Haemopoietic | 30 (2%) |
| Pancreas | 22 (2%) |
| Uncertain | 21 (2%) |
| Miscellaneous | 49 (4%) |
| | 1300 |

Disease of the cardiovascular system was the commonest cause of death in the entire group, 277 patients (21 per cent) dying from heart disease. Malignant disease was the second major cause of death, accounting for 261 fatalities (20 per cent). Carcinoma was an incidental finding in a further 64 patients thus raising the total incidence of malignancy to 25 per cent. 27·6 per cent males had malignant tumours compared with 21 per cent females. The incidence of malignancy in the 70–80 age group was 24 per cent, in the 80–90 group 29 per cent, and in the 90–100 group, 25 per cent. There is a close correlation between these findings and those of Howell and Piggot (1952) in their series of autopsies on patients dying between the age of 75 and 79. They found cardiovascular disorders foremost as a cause of death, with cancer second, and tumours of the large intestine, as in this series, the most frequent of all malignancies.

An indication of the sites most frequently involved in this series is given in Table II.

TABLE II

| Site. | Total number of tumours. | Number as cause of death. |
|---|---|---|
| Large intestine (including rectum) | 73 | 67 |
| Prostate | 47 | 27 |
| Stomach | 42 | 29 |
| Lungs | 32 | 27 |
| Oesophagus | 17 | 17 |
| Bladder | 16 | 14 |
| Kidney | 15 | 11 |
| Pancreas | 14 | 14 |

*Malignant disease of the alimentary tract*

Carcinoma of the large intestine was the commonest tumour found in this series, occurring in 73 cases and proving fatal in 67. Its great frequency contributed largely to the importance of disease of the alimentary tract as a cause of death. Males were affected more frequently than females (41 M. : 32 F.) but in view of he greater number of males than females in the series these figures actually represent a slight preponderance of this form of carcinoma in the female. In both sexes there was a relatively higher incidence in the 80–90 age group than in the 70–80 group.

In 12 cases the tumour was situated in the caecum, in 49 in the colon, and in 12 in the rectum. The pelvic colon was the most frequent site being involved in 16 cases. More than one-third of the patients died following operative treatment, 16 undergoing resection and 7 colostomy. In the unoperated group acute obstruction was a common termination occurring in 24 cases, and in 12 of these perforation and peritonitis had resulted. In 4 the perforation had developed at the site of the tumour and in 8 proximally. In colonic growths the caecum ruptured in 4 instances.

A study of metastatic spread revealed one or two interesting features. In a surprisingly high number of cases, 35/73, no metastases were discovered. However operative resection had been performed in 16 of these, 6 were incidental tumours and 10 of the 12 caecal growths showed a remarkable absence of metastases. Extensive lymph node involvement was found in 16 cases, and metastases to the liver in 15. Deposits in the lungs were discovered in only 3 cases, and in bone in 2. Carcinomatosis peritonei occurred only 3 times. One case was of particular interest in illustrating the long survival which may occur in carcinoma of the large bowel. A male of 78 years had a right hemicolectomy for carcinoma of the colon 16 years previously. A recurrence of tumour developed at the site of anastomosis and was resected 10 years later. He died 6 years after this operation with intestinal obstruction due to adhesions and no residual tumour and no metastases were found.

The findings in this group of elderly patients show that carcinoma of the large bowel most often proved fatal by reason of its local complications, frequently giving rise to obstruction with or without perforation and peritonitis. The operative mortality for resection was also high. Dissemination of the tumour was not so important a factor in causing death since less than 25 per cent of cases showed metastases in the liver, peritoneal spread was rare, and pulmonary involvement was seldom seen.

Carcinoma of the stomach was the third commonest tumour, with 42 examples, 29 proving fatal and 13 incidental. The sex ratio was 28 M.: 14 F. Thirty-five occurred in the 70–80 age group, and 7 in the 80–90 group. Only 3 patients received operative treatment, to which they all succumbed. One patient had Wernicke's encephalopathy. In the 26 fatal cases (without operation) 21 had lymph node metastases, 13 had metastases in the liver, 5 in the lungs, 3 in bone, and 8 had carcinomatosis peritonei. In the incidental groups of 13 cases only 2 showed lymph node metastases. The findings in this group differ considerably from those in the colonic group. Operative treatment was rare and was poorly tolerated. Dissemination of the tumour was more frequent and widespread. This was not entirely due to a delay in seeking treatment for in no case was the history longer than 2 years and in many, symptoms had been present for only some weeks. Another striking feature, of uncertain significance, was the presence of other major pathological conditions in many patients with gastric carcinoma, whereas the colonic group was remarkably free from other diseases. On the whole, the occurrence of so many unsuspected gastric tumours, and the degree of spread in the fatal cases in spite of a short history, would suggest that carcinoma of the stomach is often well established before symptoms arise.

Carcinoma of the oesophagus was 5th in frequency, occurring in 17 patients and proving fatal in all. The sex ratio was 14 M.: 3 F. Thirteen occurred in the 70–80 age group, and 4 in the 80–90 group. Only one tumour involved the upper end of the oesophagus, 8 the level of the bifurcation of the trachea, and 8 the distal third. The tumour remained well localised in a high percentage of cases, 11 with no metastases, 6 with lymph node metastases and only 3 with dissemination to lungs, liver, kidney and bone. Five patients died post-operatively and most of the others from local complications such as mediastinitis, bronchopneumonia and empyema. These figures do not represent the true incidence of this disease in elderly patients, as the number of cases studied depends largely on local surgical interest and other factors. There are no very accurate figures indicating its frequency in the elderly, but Howell and Piggot (1952) found it 4th in the late 70's and over 80 age group. In most recorded series the preponderance in the male sex is striking, and apart from local lymph node metastases, widespread dissemination appears to occur in only a small proportion of cases.

This short analysis of new growths of the alimentary tract indicates that in this series of elderly patients carcinoma of the large intestine was the commonest tumour encountered, gastric carcinoma was third, and oesophageal carcinoma fifth, these together constituting just over 40 per cent of all malignancies in the series.

*Carcinoma of the prostate*

Carcinoma of the prostate was the second most frequent tumour, but as a cause of death from carcinoma it ranked third. There were 47 cases of frank carcinoma, 27 of whom died as a result of the disease. Nine patients died post-operatively and in the remainder death was generally due to ascending infection and uraemia. Seven of the fatal cases were recurrences 2–8 years after prostatectomy. As regards dissemination in the fatal cases lymph node metastases were found in 8, pulmonary in 6, hepatic in 2, and bone in 6. Of the remaining 20 incidental tumours wide infiltration of the gland by carcinoma was noted, often with perineural lymphatic and lymph node involvement.

In addition to these 47 examples of carcinoma of the prostate, there was focal carcinomatous change in another 38 cases, which brings the total incidence of prostatic carcinoma in the series to 11 per cent. An even higher incidence (14 per cent) was noted by Rich (1935) in 292 consecutive autopsies on men over the age of 50, and Moore (1935) discovered prostatic carcinoma with a frequency which increased with age, mounting to 29 per cent, in the 9th decade. An increase with age was also experienced in this series, with 4·6 per cent of tumours occurring in the 70–80 age group, compared with 14·6 per cent in the 80–90 group. This confirms Willis' statement (1953) that most deaths from this disease occur in the 8th decade.

*Carcinoma of the lung*

Carcinoma of the lung was the 4th commonest tumour, there being 32 cases. It accounted for 27 deaths. As in all recorded series the sex ratio was predominantly male, 22 M.: 10 F. Twenty-five cases occurred in the 70–80 age group, 6 in the 80–90 group, and 1 in a patient aged 90. Considering the numbers in each group there was no waning incidence with age though in most series the peak has been noted to occur in the 6th decade. Five of the tumours were incidental and in addition to these, 5 microscopic carcinomas were found in scars. There was also 1 pleural endothelioma. Histologically 16 were oat cell, 10 were squamous, and 6 adenocarcinoma. Apart from hilar node involvement which was noted in more than three-quarters of the cases, the liver contained metastases in 13, adrenals in 6, and bone and brain in 5.

*Other tumours*

Other tumours which occurred with some frequency were carcinoma of the bladder, kidney and pancreas. There were 16 malignant bladder tumours, all of squamous type except for 1 round-cell sarcoma. Males predominated with a 12 M. : 4 F. incidence. Fourteen tumours occurred in the 70–80 age group, and 2 in the 80–90 group. The cause of death was chiefly ureteric obstruction with ascending infection and uraemia. Three patients died shortly after ureteric transplantation and 1 survived a year. Distant metastases occurred in only 2 cases.

Of the 15 malignant tumours of the kidney, 12 were hypernephromas, 2 carcinomas of the pelvis, and 1 myosarcoma. One of the pelvic tumours had eroded the wall of the duodenum establishing a wide fistulous communication, with duodenal contents passing freely into the renal pelvis. Four of the hypernephromas were incidental findings, and one an extremely small tumour only 1 cm. in size. Again males predominated, 11 M.: 4 F. Pulmonary metastases were found in only 2 cases and vertebral and epidural space in 1.

There were 14 cases of pancreatic carcinoma, fatal in all. The sex incidence was 9 M.: 5 F. Twelve of the tumours were situated in the head, 1 in body and tail, and 1 in tail. Nine patients had jaundice and there was a very high incidence of metastases, with liver involvement in 11, and pulmonary in 8. Death was due to pulmonary embolism from peripheral venous thrombosis in 2 cases, and in a third case the popliteal and femoral veins showed thrombosis secondary to actual tumour invasion of their walls.

Of the remaining tumours there were 8 examples of carcinoma of the breast, 1 occurring in a male, and 7 uterine tumours of which 5 were carcinomas and 2 sarcomas. There were 8 malignant tumours arising in the bile ducts, and 7 in the gall bladder. This was the only growth common to both sexes which showed a marked predominance in the female (5 F.: 2 M.). Carcinoma of the liver was encountered 4 times, in each instance superimposed on nodular hyperplasia. Brain tumours were infrequent only 4 glioblastomas being found, all in males, and 1 astrocytoma in a female. There were 4 cervical carcinomas, 2 of vulva, 1 of ovary, 1 of thyroid, 1 of larynx and 1 of adrenal cortex. Reticulum sarcoma and lymphosarcoma were each encountered twice, and retroperitoneal sarcoma and melanoma once. There were 8 examples of lymphatic leukaemia, and 2 of multiple myeloma. No tumour of testis or of bone was found in the series.

*Multiple malignancies*

The frequency of multiple malignancies in the same patient was not striking. Apart from focal carcinoma of the prostate of which there were 11 cases associated with malignant tumours in other sites there were only 4 genuine examples: carcinoma of the lung and bladder, myosarcoma of the kidney and carcinoma of the stomach, glioblastoma and carcinoma of the stomach, hypernephroma and carcinoma of stomach. Lymphatic leukaemia occurred in association with carcinoma of the lung in 1 case, and with an extensive carcinoma of the prostate in another.

### CONCLUSIONS

In this series of 1300 post mortems on patients dying over the age of 70, heart disease ranked first and cancer second as a cause of death. Twenty-five per cent of patients in the series had malignant disease and this proved fatal in 20 per cent. Males were affected more frequently than females, confirming the findings of other workers on the sex incidence of carcinoma in later life. The incidence of tumours rose slightly in the 8th decade as compared with the 7th, but this was partly accounted for by the increasing incidence of carcinoma of the prostate with age.

The commonest site for a malignant tumour was the large intestine, 22 per cent of all tumours occurring in this situation. Metastatic spread was a much less marked feature of this tumour than of carcinoma of the stomach. The second most common tumour was carcinoma of the prostate, accounting for 14 per cent of all malignancies. When focal carcinomatous change was included, the total incidence of malignant disease of the prostate rose to 11 per cent of the males in the series.

Tumours of the stomach, lungs, oesophagus, bladder, kidney and pancreas followed in that frequency, and tumours which were rare were those of cervix and ovary, thyroid, and melanoma. Nevertheless most sites were represented but tumours of bone and testis were not encountered. The incidence of multiple malignancies in the same patient was not striking, only 4 examples being found.

Of all the malignant tumours discovered 20 per cent were incidental in that they were unsuspected and did not contribute materially to the death of the patient. Such a finding emphasises the importance of complete post mortem examination as the only means of determining the true incidence of any tumour.

Thanks are due to the Medical Staff of the City and Royal Victoria Hospitals, Belfast, for access to the clinical records, and to the Northern Ireland Hospitals Authority for a research grant to this Department.

### REFERENCES

Howell, T. H. and Piggot, A. P.—(1951) *Geriatrics*, **6**, 85.—(1952) *Ibid.*, **7**, 137.
Medalia, L. S. and White, P. D.—(1952) *J. Amer. med. Ass.*, **149**, 1433.
Moore, R. A.—(1935) *J. Urol.*, **33**, 224.
Rich, A. R.—(1935) *Ibid.*, **33**, 215.
Willis, R. A.—(1953) ‘ Pathology of Tumours.’ London (Butterworths), p. 586.